# Hahnemann and Ziemssen

**Published:** 1884-07

**Authors:** 


### HAHNEMANN AND ZIEMSSEN.

Hahnemann tells us in the *Organon* (fifth edition, page 84, §45) * * * "Two diseases, though different in kind but very similar in their manifestation of suffering and symptoms, will always extinguish each other whenever they meet in the organism; the stronger disease will overcome the weaker one." * * * As corroborative of this we quote the following from Ziemssen's *Cyclopædia of the Practice of Medicine* (Vol. II, p. 469):

"A peculiar effect is often exercised by *erysipelas* upon pre-existing chronic or acute morbid processes in the *skin*. Cazenave, Schedel, Sabatier, and others have observed that after the termination of a casual attack of erysipelas, *old* eczemas, lupous affections, and ulcers upon the legs are quickly healed. In several cases reported by Despres, Champouillon, Mauriac, and others, extensive phagedæmic and serpiginous chancres, and even chronic abscesses, produced by caries of the bones, which had previously defied all treatment, healed rapidly during an erysipelas."

At § 38 (top of p. 80, fifth edition) Hahnemann writes:

"Whenever two dissimilar diseases meet in the body, the stronger one always suspends the weaker (provided they do not combine, which seldom happens in the acute forms), *but they never cure each other.*"

To illustrate this point, Hahnemann cites the useless practice of forming setons, etc., to cure internal troubles. He says the disease "is only now and then rendered dormant * * * when the irritation created by the setons is more intense than the internal disease."

Ziemssen writes:

"Even extensive non-malignant tumors can be made to undergo absorption, according to the observations of Legrand, Busch, and Volkmann, by erysipelas, *although the cure does not* seem to be *always permanent.*" (Italics ours.)

Now it can scarcely be denied that erysipelas is at least "very similar in its manifestations of suffering and symptoms" to eczemas, ulcers, and abscesses. And it *cures them.* On the contrary, it cannot be claimed that erysipelas closely resembles *tumors,* which we are told it does not cure but relieves, the dissimilar masking the one the other.

